# Efficacy and safety of azithromycin in preventing bronchopulmonary dysplasia in preterm infants: a systematic review and meta-analysis

**DOI:** 10.3389/fped.2026.1780809

**Published:** 2026-03-24

**Authors:** Fatima Albeladi, Maya Essam Alhindi, Raghad Essam Marghalani, Tassnim Sherif Hafez, Joud Mohammed Aljuhani, Arwa Mohammed Mahmud Alqadi, Abdulaziz Faisal Alahmadi, Jana Muqbil Alahmadi, Rawan Faisal Alahmed, Reema Mubarak Alanazi, Riham Yousef Tashkandi, Latifa Hani AlAmiri, Raghad Mansour Nawar Aljuaid, Turki Alahmadi

**Affiliations:** 1 College of Medicine, University of King Abdulaziz, Rabigh, Saudi Arabia; 2 College of Medicine, Taif University, Taif, Saudi Arabia; 3 College of Medicine, University of King Abdulaziz, Jeddah, Saudi Arabia; 4 College of Medicine, Almaarefa University, Riyadh, Saudi Arabia; 5 College of Medicine, Umm Al Qura University, Mecca, Saudi Arabia; 6 College of Medicine, Al-Rayyan Colleges, Madinah, Saudi Arabia; 7 College of Medicine, Taibah University, Madinah, Saudi Arabia; 8 College of Medicine, King Faisal University, Al-Ahsa, Saudi Arabia; 9 College of Medicine, Northern Border University, Arar, Saudi Arabia; 10 Department of Pediatrics, Faculty of Medicine, King Abdulaziz University, Jeddah, Saudi Arabia

**Keywords:** azithromycin, bronchopulmonary dysplasia, meta-analysis, preterm infants, prophylactic azithromycin, systematic review, therapeutic azithromycin, Ureaplasma

## Abstract

**Introduction:**

Ureaplasma colonization in preterm infants is linked to pulmonary inflammation and bronchopulmonary dysplasia (BPD). Evidence regarding the effectiveness of macrolides, including their prophylactic role in preventing BPD, remains limited. This review aimed to assess whether azithromycin reduces the incidence of BPD or death in preterm infants, with or without Ureaplasma colonization, compared with placebo or no intervention.

**Methods:**

We followed PRISMA guidelines and searched PubMed, Cochrane Library, Embase, and Google Scholar for randomized controlled trials (RCTs) evaluating azithromycin in preterm infants with unknown or confirmed Ureaplasma infection. Primary outcomes were death and BPD, while secondary outcomes included hospital stay, adverse events, and ventilation or oxygen duration. Studies not focused on preterm infants, lacking Ureaplasma status, or not randomized were excluded.

**Results:**

A meta-analysis of six randomized controlled trials, including 1,446 preterm infants with unknown or confirmed Ureaplasma status and sample sizes ranging from 80 to 796, compared azithromycin with placebo. No significant reduction was observed in BPD incidence [risk ratio (RR) = 0.92, 95% CI: 0.78–1.09, *p* = 0.35] or mortality (RR = 0.88, 95% CI: 0.66–1.18, *p* = 0.39). Heterogeneity was low for mortality (*I*^2^ = 0%) and moderate for BPD (*I*^2^ = 61%). Secondary outcomes showed no significant differences in mechanical ventilation days (mean difference=0.07, 95% CI: −0.78 to 0.33, *p* = 0.58), supplemental oxygen requirement (mean difference=−0.45, 95% CI: −0.98 to 0.07, *p* = 0.09), or hospitalization duration (mean difference=−0.02, 95% CI: −0.19 to 0.15, *p* = 0.85). Heterogeneity (*I*^2^) was 94% for supplemental oxygen, 71% for mechanical ventilation, and 0% for hospitalization duration. Risk-of-bias assessment identified two studies with low risk and four with unclear risk, indicating acceptable methodological quality.

**Conclusion:**

This systematic review and meta-analysis evaluated azithromycin efficacy and safety in preventing BPD and reducing mortality in preterm infants. No significant reduction in BPD or mortality was observed with azithromycin compared with placebo. Secondary outcomes, including ventilation duration, supplemental oxygen use, and hospitalization length, showed no improvement. However, heterogeneity among secondary outcomes was moderate to high, and included studies were limited.

**Systematic Review Registration:**

PROSPERO CRD42024572847.

## Introduction

The development of pulmonary inflammation and congenital abnormalities in premature newborns has been linked to Ureaplasma spp (1). In preterm newborns, bronchopulmonary dysplasia (BPD) remains a serious health issue that can have an impact on future growth and neurodevelopment, as well as increase medical expenses and prolong hospitalizations (2). Additionally, it is characterized by parenchymal fibrosis, neutrophilic inflammation, smooth muscle hypertrophy, and widespread airway injury (3). Several factors lead to BPD, but the main cause is impaired lung growth *in utero*, which makes the lungs of preterm infants more susceptible to damage from oxygen therapy or assisted ventilation (4, 5).

There is a significant lack of strong evidence regarding the effectiveness of macrolides in treating Ureaplasma colonization and their prophylactic use in preventing BPD in preterm infants regardless of Ureaplasma culture status (6). Despite being recognized for their notable anti- inflammatory properties, macrolides, particularly intravenous azithromycin (AZM) have been evaluated in several pilot randomized clinical trials for their efficacy and safety in preterm infants with extremely low gestational age (7). Studies have found that a 3-day course of AZM can effectively eliminate Ureaplasma in this population (8), supporting its potential use in interrupting the inflammatory cascade implicated in BPD. AZM is increasingly viewed as a promising intervention for chronic inflammatory conditions, particularly BPD in preterm neonates (9). However, current literature remains limited by small sample sizes, variability in dosing protocols, and inconsistent inclusion criteria, especially concerning Ureaplasma status (6, 7, 9). While some systematic reviews and pilot studies have reported a reduction in the incidence of BPD with macrolide use (7, 9), the findings are still inconclusive, and long-term safety data are sparse (6, 8). As such, although AZM shows potential as both a prophylactic and therapeutic agent, its routine use for BPD prevention is not yet supported by robust, large-scale randomized controlled trials (6, 7). This review aimed to evaluate the efficacy and safety of prophylactic or therapeutic AZM in reducing the incidence of BPD or death in preterm infants with unknown or proven Ureaplasma status compared with placebo or no intervention.

## Material and methods

### Review of the literature

In our review, we followed the Preferred Reporting Items of Systematic Reviews and Meta- Analyses (PRISMA) model to ensure the inclusion of studies with minimal bias (10). The study protocol was registered in PROSPERO *a priori* with the following ID: CLRD42024572847 (11). Ethical approval was not required because of the nature of this study. In July 2024, we conducted a systematic search of the following databases: (1) PubMed, (2) Google Scholar, and (3) Web of Science in july 2024, with the final search completed on that date. A search was conducted using the following keywords: (“azithromycin” OR “prophylactic azithromycin”) AND (“bronchopulmonary dysplasia” OR “BPD”). Studies were evaluated for inclusion based on Population, Intervention, Comparison, Outcome, Timing, and Setting (PICOTS) criteria. The population comprised preterm infants with either unknown or confirmed Ureaplasma status. The intervention involved administration of AZM for either prophylactic use in at-risk infants prior to confirmation of infection or therapeutic use in those with suspected or confirmed infection. The comparison group received either placebo or no treatment. The primary outcomes of the study were BPD and mortality, while secondary outcomes included the duration of supplemental oxygen or ventilation, hospital stay length and incidence of adverse events. Only randomized RCTs were included in the study. Data extraction, analysis, and manuscript preparation were subsequently completed, and the study was finalized in November 2025.

### Methodology for selecting studies

Involved studies in our systematic review and meta-analysis had to meet the following criteria: (1) written in English, (2) publications without timeframe limitations, (3) studies that reported the number of preterm infants (<37 weeks gestational age) with unknown or proven Ureaplasma status, (4) studies that reported the number of preterm infants receiving prophylactic or therapeutic AZM compared to placebo or no intervention, (5) randomized controlled trials, (6) studies reporting outcomes of interest relevant to the clinical questions, such as the primary (BPD, death) and secondary outcomes (duration of mechanical ventilation or supplemental oxygen, length of hospital stay, and incidence of adverse events). Studies were excluded from our systematic review based on the following criteria: (1) studies that did not investigate AZM; (2) studies that did not focus on preterm infants or those without specific mention of Ureaplasma status; (3) non-randomized studies, case reports, and reviews; (4) studies that lacked a placebo or did not have an intervention control group; (5) studies not reporting outcomes of interest for clinical questions, such as BPD, death, or secondary clinical outcomes; and (6) studies that had been screened for duplication.

### Process of screening and data extraction

Independent reviewers (AF, AME, ARM, and ALH) screened the papers simultaneously and independently reviewed them by title and abstract using the Rayyan search web application for systematic reviews (12). Next, the full texts of the articles were reviewed by five independent reviewers (AF, AME, AJM, ARM, and TRY) simultaneously, with any differences resolved by discussion. Afterwards, data extraction was performed by four reviewers (AME, AAF, AJM, and HTS) for the following variables: (1) total number of patients, (2) number of patients in the intervention group, (3) number of patients in the control group, (4) mean age of the patients in weeks, (5) age range of patients, (6) outcomes measured, (7) comorbidities, (8) birth weight of the neonates, (9) Apgar score, (10) Ureaplasma status, (11) sepsis, (12) other medications, (13) follow-up duration in months, (14) type of intervention and administration, (15) comparison group details of intervention, (16) dosage of AZM, (17) duration of AZM, (18) incidence of BPD, (19) incidence of death, (20) duration of mechanical ventilation, (21) duration of supplemental oxygen, (22) duration of hospital stay, (23) adverse events, (24) measurement tools, (25) time points of measurement, (26) statistical methods, effect size, (28) confidence intervals, (29) heterogeneity, (30) publication bias, (31) main findings, (32) limitations, (33) number of male patients, (34) number of female patients, and (35) recommendations for future research.

### Assessment of quality and bias risk

Five investigators (AF, AME, ARM, AJM, and TRY) assessed the quality of the studies using the Cochrane Risk of Bias tool (RoB 2) to assess the risk of bias in randomized trials (13). Methodological quality was evaluated by determining selection bias (random sequence generation and allocation concealment), performance bias (blinding of participants and personnel), attrition bias (incomplete outcome data), detection bias (blinding of outcomes assessment), reporting bias, and other possible sources of bias. Studies were considered to have a low risk of bias if they were at a low risk for four or more domains. Other studies were classified as high risk. Disagreements were resolved by discussion.

### Publication bias

Due to the limited number of included studies (<10), a formal assessment of publication bias using funnel plots or statistical tests was not performed, in accordance with current methodological guidelines.

### Statistical analysis

RevMan software, version 5.1.2, was used to analyze the data. For categorical outcomes, RRs with 95% CIs were constructed using a random-effects model. The Q test and I° statistics were used to analyze study heterogeneity and categorize it into four quartiles: low, moderately low, moderately high, and high. The classification of each category was based on intervals: 0% to <25%, 25%–50%, 50%–75%, and ≥75%. The 95% confidence intervals for the data are displayed as forest plots. Statistical significance was set at a *P*-value <0.05.

## Results

**Figure 1:**
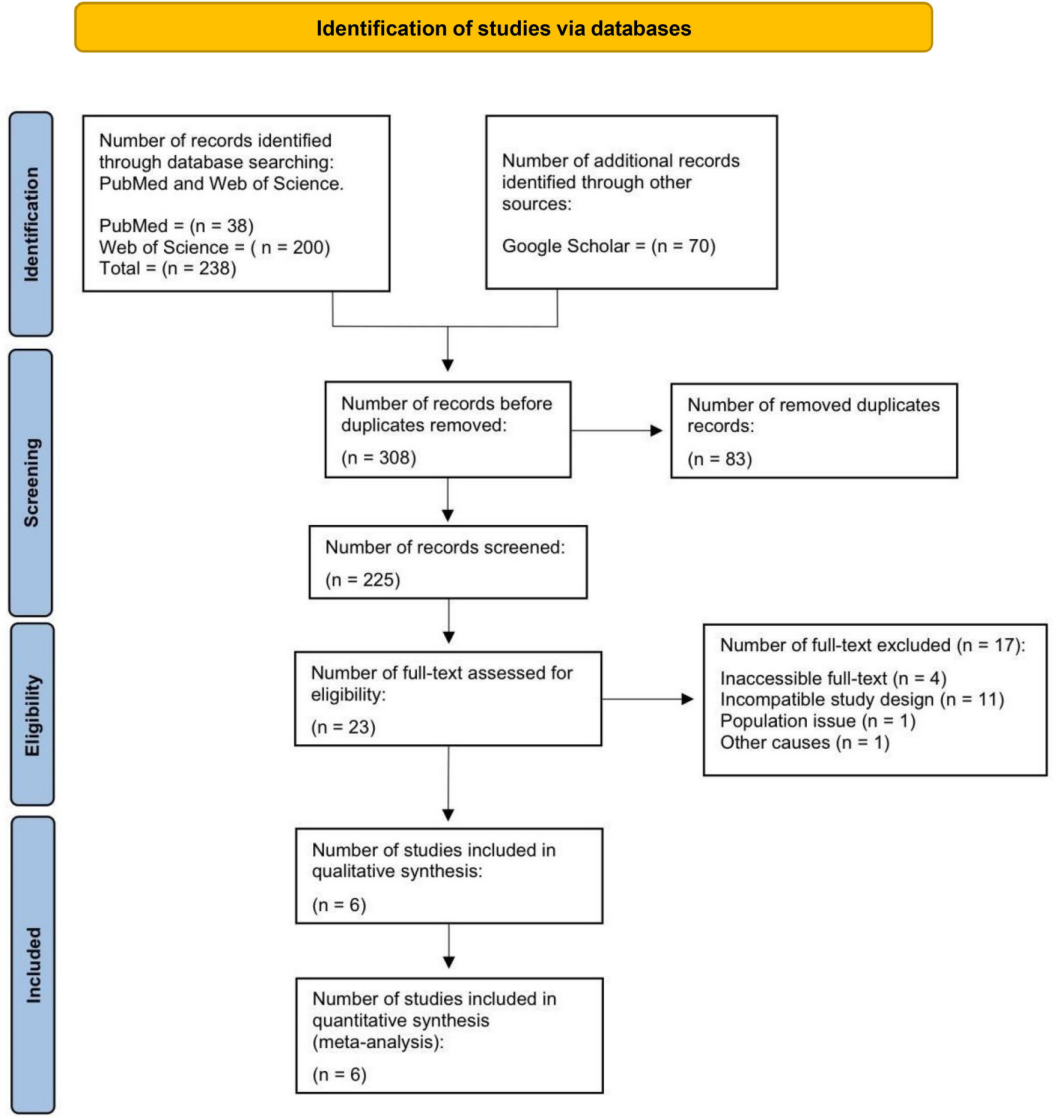
PRISMA flow diagram.

The literature search generated 308 articles from Google Scholar (70), Web of Science (38), and PubMed (200) databases. After excluding 83 duplicate articles, 225 remained for the abstract and title screening. Two hundred and two publications were excluded because they did not meet the inclusion criteria. The 23 articles remaining were evaluated to determine their eligibility for full-text review. Additionally, 17 articles were excluded for various reasons, such as inaccessible full-texts, incompatibility of the study design, and population issues. Finally, six publications that satisfied the predetermined criteria were included. All six studies were incorporated into both the qualitative and quantitative syntheses (*n* = 6) (8, 14–18) (See Figure 1). Table 1 summarizes the articles included in this review. All studies examined how preventive or therapeutic AZM reduced the likelihood of BPD or mortality in preterm infants with unknown or confirmed Ureaplasma status compared to a placebo. Every study was conducted in a country or region worldwide and published in English. The studies included were conducted in three regions of the United States, one in the United Kingdom, one in Turkey, and one in Brazil. The selected studies had a total sample size of 1,446 respondents, where the study with the smallest sample had 80 participants, and the largest study had 796 participants.

**Table 1:** Characteristics of the included studies.

Author	Region	Study design	Sample size	Intervention	Inclusion	Results	Conclusion
Viscardi et al., 2022 (8)	United States	Randomized Controlled Trial (RCT)	121	Intravenous azithromycin	ELGAN newborns between the ages of 24 and 28 weeks were enrolled.	Although there were no statistically significant differences in mortality or severe respiratory morbidity between the azithromycin and placebo groups, infants who tested positive for *Ureaplasma* through tracheal aspiration had a greater incidence of death or severe respiratory morbidity between the ages of 22 and 26 months.	Azithromycin treatment in the first week of life did not significantly alter the long-term pulmonary and neurodevelopment outcomes of preterm newborns.
Ballard et al., 2011 (14)	United States	Randomized Controlled Trial (RCT)	220	Intravenous azithromycin	Among the requirements for enrollment were 1,250 grams at birth, 12 h of intermittent artificial ventilation, and 72 h of age.	An odds ratio of 0.46 indicated that the azithromycin group had a lower mortality and BPD incidence than the placebo group. This, however, was not statistically significant. In the *Ureaplasma* subgroup, the azithromycin group similarly has shown a reduction in BPD or death.	Routine azithromycin therapy is not advised for the prevention of BPD. Patients with *Ureaplasma* colonization may benefit from early treatment, but a bigger trial is required.
Nunes et al., 2020 (15)	Brazil	Randomized Controlled Trial (RCT)	80	Intravenous azithromycin	Newborns with very low birth weights who were admitted to the intensive care unit (NICU) and who were given invasive mechanical ventilation within 72 h after delivery, as well as invasive ventilation support for a minimum of 12 h.	Notwithstanding the presence of *Ureaplasma* in the blood, azithromycin treatment significantly decreased serum IL-2 and IL-8 levels, decreased mortality, and decreased O_2_ dependency in 40 patients.	Azithromycin has anti-inflammatory qualities; it reduces cytokines after five days, and in preterm neonates on mechanical ventilation, it reduces death and O_2_ dependency at 28 days/death.
Viscardi et al., 2020 (16)	U.S. Maryland state - Baltimore	Randomized Controlled Trial (RCT)	121	Azithromycin antibiotic drug	Babies with extremely low gestation (ELGAN) between weeks 240 and 286 who were less than 72 h postnatal and receiving positive pressure breathing for at least one hour were included.	After evaluating 121 infants’ *Ureaplasma*-free survival, the study noted that azithromycin was the most successful treatment. Nevertheless, 84% tested positive for *Ureaplasma*, which increased infant death and morbidity.	In this trial, a 3 days azithromycin treatment successfully eliminated *Ureaplasma* colonization in the respiratory tract.
Gharehbaghi et al., 2012 (17)	Turkey	Randomized Controlled Trial (RCT)	108	Oral azithromycin, vitamin A, aminophylline,	Preterm newborns under 32 weeks gestation weighing 1,500 gram at birth who were admitted to the NICU for enrollment were eligible for our study.	The two groups’ gestational ages and participant sex distributions did not differ significantly. At 28 days, 25% of patients in the azithromycin group had BPD, compared to 43% of control patients.	Azithromycin effectively lowers the incidence of BPD in low birthweight infants. However, more extensive research is required before it can be used routinely.
Lowe et al., 2024 (18)	United Kingdom	Randomized Controlled Trial (RCT)	796	Antibiotic therapy	The trial was open to infants under 30 weeks of gestation with at least 2 h of invasive or non-invasive breathing support within 72 h of birth. Additionally, they required written informed consent, feasible follow-up, and a high likelihood of an indwelling intravenous line for 10 days.	Pulmonary *Ureaplasma* spp colonization did not affect survival; 42% of 394 infants in the intervention group and 45% in the placebo group survived without moderate or severe CLD.	Azithromycin used prophylactically, independent of *Ureaplasma* spp colonization, does not increase survival without physiologically characterized CLD.

NICU, neonatal intensive care unit; RCT, randomized controlled trial, CLD, chronic lung disease of prematurity; BPD, bronchopulmonary dysplasia; ELGAN, extremely low gestation newborns; IL, interleukin; O_2_, oxygen.

### Meta-analysis results

**Figure 2:**
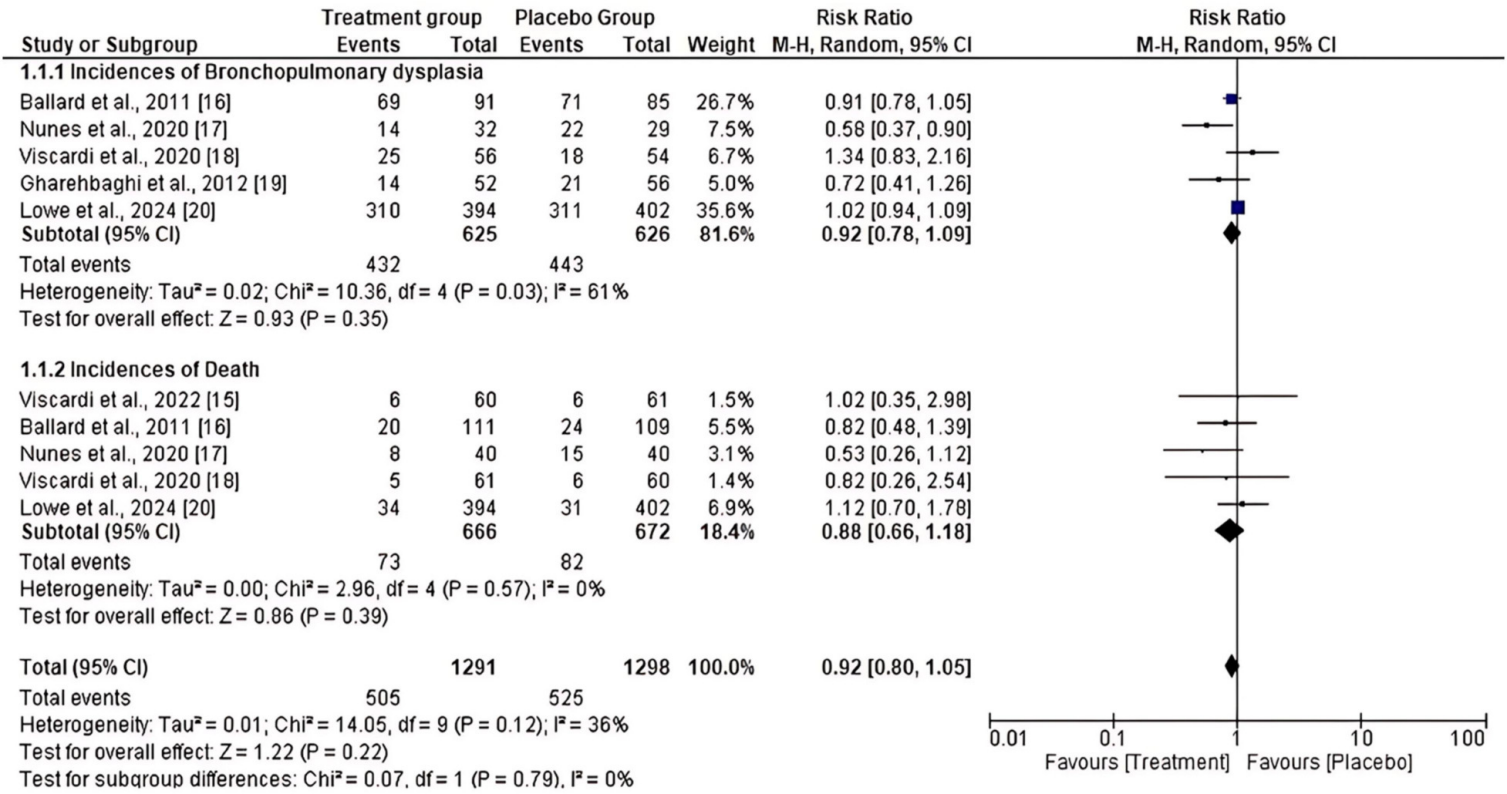
Forest plot of primary outcome effect of prophylactic azithromycin treatment.

**Figure 3:**
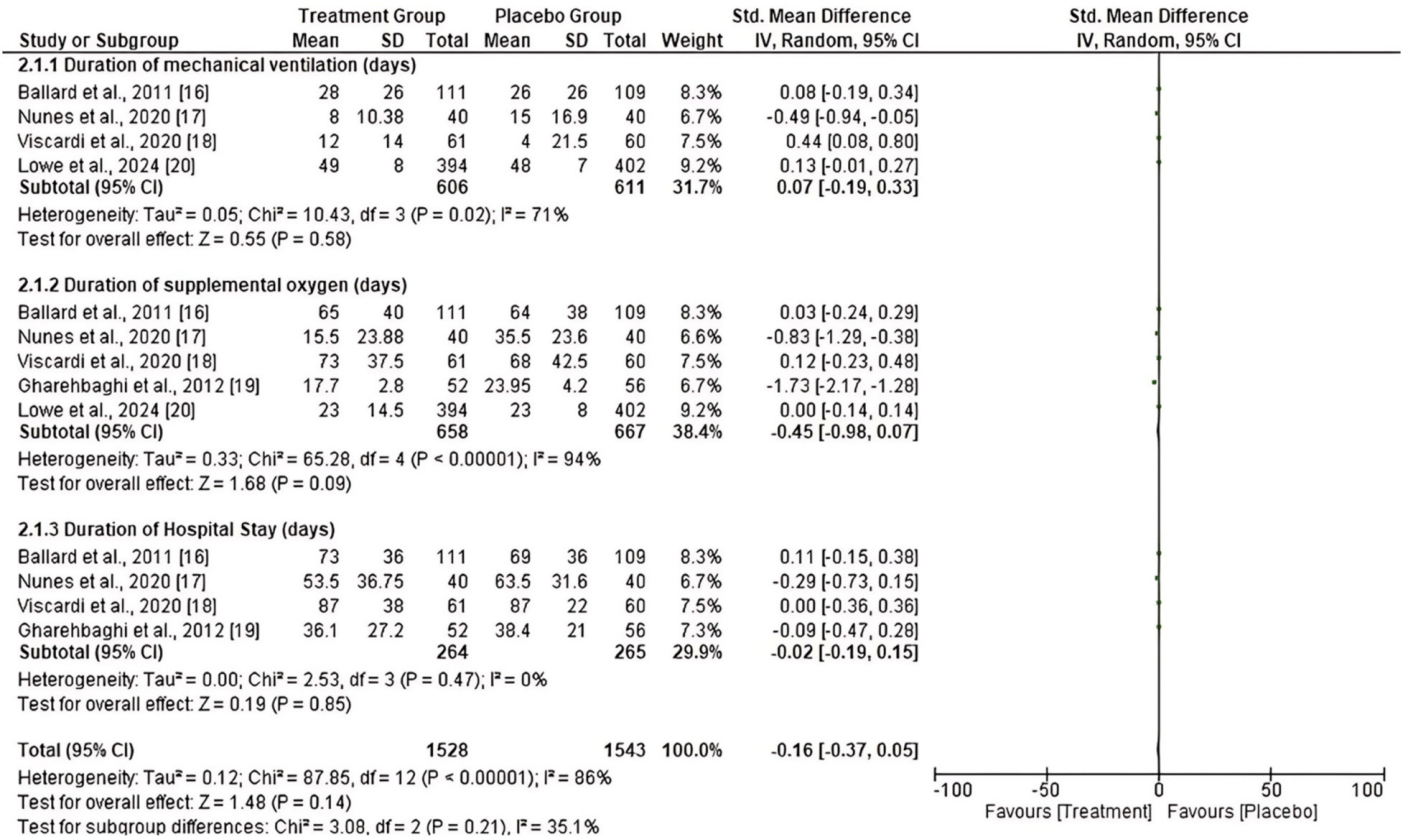
Forest plot of secondary outcome effect of prophylactic azithromycin treatment.

A meta-analysis was performed to assess the efficacy and safety of prophylactic or therapeutic AZM in decreasing the incidence of BPD or death in preterm infants with unknown or confirmed Ureaplasma status compared to placebo. The primary and secondary results of the included studies were analyzed. The primary outcome results showed moderately high heterogeneity among the five studies that assessed the incidence of BPD (*I*^2^ = 61%). The heterogeneity of the five studies that assessed the incidence of death was very low (*I*^2^ = 0%). Nonetheless, the total number of events in the treated group was lower compared to the placebo group (505 vs. 525). The results showed no significant decrease in the incidence of BPD [RR = 0.92, 95% CI (0.78, 1.09)] or death [RR = 0.88, 95% CI (0.66, 1.18)] between the AZM and placebo groups. The *p*- values for the two factors assessed were 0.35 and 0.39, respectively (Figure 2). In addition, the secondary outcome results showed moderately high heterogeneity of the four studies assessing the duration of mechanical ventilation (d) (*I*^2^ = 71%). In comparison, the five studies evaluating the duration of supplemental oxygen (d) showed high heterogeneity (*I*^2^ = 94%). However, the four studies that assessed the duration of hospital stay (d) showed a very low heterogeneity (*I*^2^ = 0%). However, the results showed that there was no statistically significant reduction in the duration of mechanical ventilation (d) [MD = 0.07, 95% CI (−0.78, 0.33), *P* = 0.58], duration of supplemental oxygen (d) [MD = −0.45, 95% CI (−0.98, 0.07), *P* = 0.09] and the duration of the hospital stay (d) [MD = −0.02, 95% CI (−0.19, 0.15), *P* = 0.85] between the group treated with AZM and the placebo group. However, the overall duration in the treated group was lower than that in the placebo group (Figure 3).

### Risk of bias assessment

**Figure 4:**
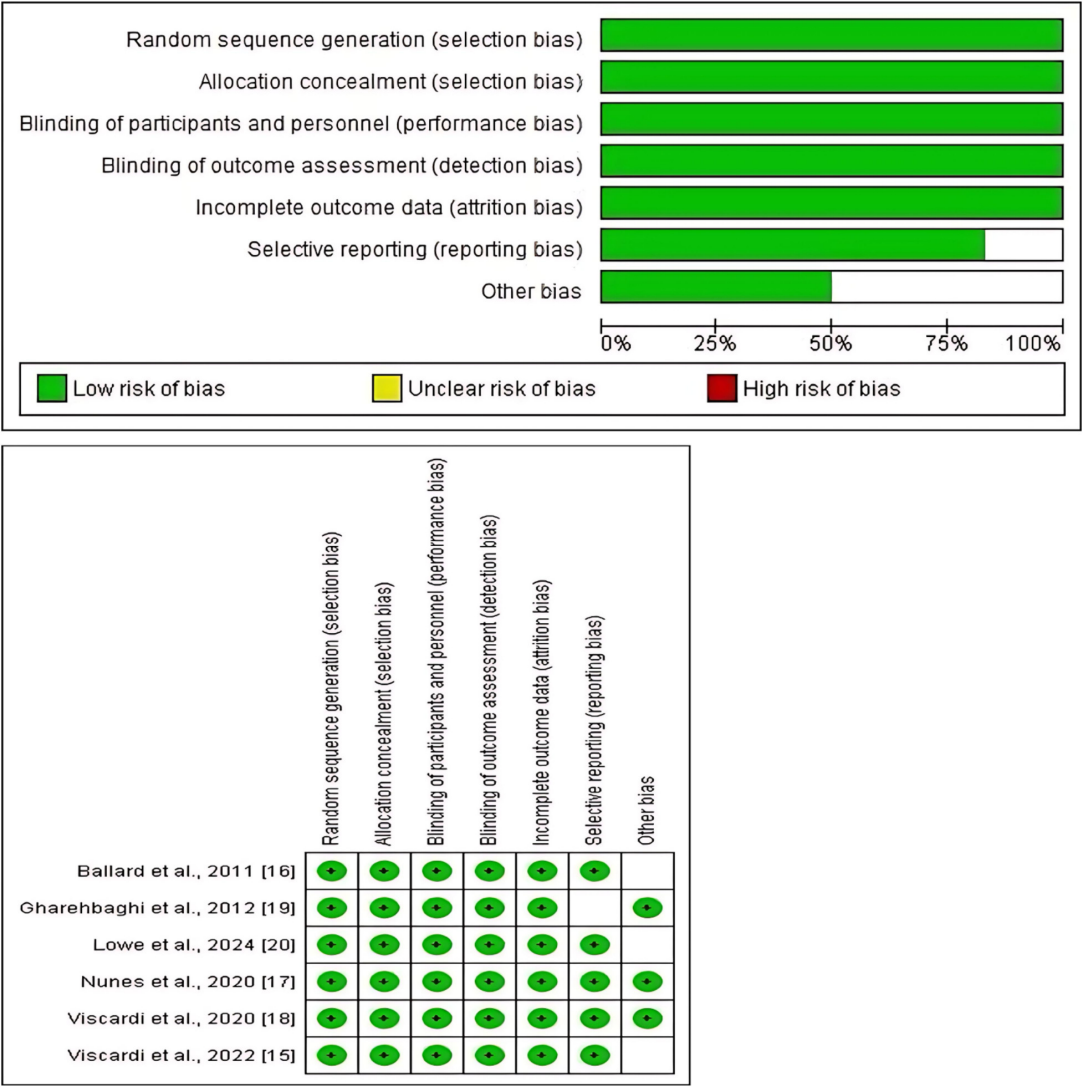
Cochrane risk of bias assessment of the items in the included studies.

This systematic review and meta-analysis included six studies, all of which were RCTs. We assessed the risk of bias in the included RCTs using the Cochrane Collaboration RoB 2 tool. Our findings showed that two studies had a low risk of bias, none had a high risk, and four had an unclear risk of bias. The most common sources of bias were other biases and selective reporting (reporting bias). Overall, the included studies were of acceptable methodological quality and had a low risk of bias (Figure 4).

## Discussion

AZM has been shown in some studies to reduce Ureaplasma colonization; however, the clinical implications of this finding for BPD prevention remain uncertain. Furthermore, several studies have reported reduction in the incidence of BPD in infants who received AZM compared with those who did not. Specifically, AZM was associated with decreased duration of mechanical ventilation and supplemental oxygen, contributing to a shorter hospital stay for infants.

This study aimed to determine whether prophylactic or therapeutic AZM is effective and safe for reducing the incidence of BPD or death in preterm infants, regardless of their Ureaplasma status, compared with placebo or no intervention. In addressing this aim, the present study contributes to the limited evidence regarding the potential of AZM as a therapeutic option for preterm infants at risk of BPD. In 2014, Nair et al. carried out a systematic review which showed that AZM therapy led to a significant reduction in BPD based on a meta-analysis of three RCTs (7). However, these findings were later challenged by Abdul Razak's 2020 meta-analysis, which included five RCTs (19). This review did not find any statistically significant differences in BPD, mortality, or the combined outcomes of both among preterm infants receiving AZM treatment. These results align with our findings, reinforcing the conclusion that prophylactic AZM does not significantly affect critical outcomes in this population.

A key difference between our review and Abdul Razak's meta-analysis was the scope of the data analyzed. Our review included six RCTs involving 1,446 preterm infants, whereas Abdul Razak's analysis incorporated five RCTs with 564 preterm infants. This larger dataset improves the reliability of our conclusions by drawing on a broader evidence base.

Razak et al. also emphasized the potential role of longer AZM regimens. They noted that Nair et al.'s earlier meta-analysis, which used a fixed-effects model, might have underestimated the variability between studies. In contrast, Abdul Razak's study and our review employed a random-effects model that is better suited for capturing differences across trials. This methodological approach likely provides a more accurate evaluation of AZM's actual impact on outcomes like BPD and mortality in preterm infants.

Comparing our findings with those of Abdul Razak et al., we observed some differences. Razak et al. found a significantly reduced incidence of BPD and death in Ureaplasma-positive preterm infants treated with AZM. Additionally, they reported that AZM reduced the duration of supplemental oxygen in preterm infants, regardless of their Ureaplasma status. However, these findings were not observed in the present study.

Although our review found no significant differences in BPD, death, or related outcomes in preterm infants treated with AZM, a 2020 RCT reported anti-inflammatory effects and reduced oxygen dependency at 28 days (16). However, this contrasts with a larger multicenter RCT conducted in 2024, which showed no improvement in survival without moderate or severe BPD at 36 weeks postmenstrual age (PMA) (19). The discrepancies between these studies likely stem from differences in the study populations, sample sizes, treatment regimens, and outcome measures. The 2020 study targeted a smaller, high-risk group of mechanically ventilated neonates (<1,500 g) using a 5-day 10 mg/kg/day AZM regimen. In contrast, the 2024 study included a broader group of preterm infants (<30 weeks gestation) and employed a longer 10-day regimen with a higher initial dose of 20 mg/kg/day followed by 10 mg/kg/day. Additionally, the 2020 study focused on short-term outcomes, such as oxygen dependency and cytokine levels. In contrast, the 2024 trial assessed long-term outcomes, such as survival without moderate or severe BPD at 36 weeks PMA.

### Limitations and recommendations

The primary limitation of our analysis is the limited number of contributing RCTs and the variability in their methodologies. Most included RCTs had small sample sizes, with the exception of one study (19). Differences in AZM dosage, route of administration, treatment regimens, and treatment duration contributed to heterogeneity across studies. Some heterogeneity values (*I*^2^) were high, particularly for secondary outcomes such as supplemental oxygen requirement (*I*^2^ = 94%); however, no subgroup or sensitivity analyses were conducted, which is acknowledged as a limitation. Only studies published in English and accessible through selected databases were included, which may have introduced selection bias. Additionally, due to the small number of included studies (<10), a formal assessment of publication bias using funnel plots or statistical tests was not performed in accordance with current methodological guidance, which may limit the ability to detect potential bias in study reporting. Although AZM was evaluated for efficacy, the absence of a clear clinical benefit limits the applicability of these findings to routine clinical practice. From an antibiotic stewardship perspective, the routine prophylactic use of AZM in preterm infants should be interpreted with caution, particularly in light of the limited long-term safety data and the lack of evidence regarding antimicrobial resistance.

For future research, larger RCTs evaluating AZM for BPD prevention are warranted, as well as studies assessing other macrolides. Greater consistency in dosing regimens and outcome definitions may improve comparability across studies. International collaboration may also help address language and accessibility barriers and strengthen the evidence base for future analyses.

## Conclusion

The primary goal of this systematic review and meta-analysis was to assess the efficacy and safety of AZM in preventing BPD and decreasing preterm infant mortality. According to findings from six randomized controlled studies, AZM did not decrease mortality or incidence of BPD in comparison with a placebo. Our study was limited by the small number of trials, variations in treatment regimens, and methodological variability. Further studies are required to assess the efficacy of other macrolides, and conduct large-scale RCTs examining AZM therapy to prevent BPD.

## Data Availability

The original contributions presented in the study are included in the article/Supplementary Material, further inquiries can be directed to the corresponding author.
